# Jointly Optimizing Resource Allocation, User Scheduling, and Grouping in SBMA Networks: A PSO Approach

**DOI:** 10.3390/e27070691

**Published:** 2025-06-27

**Authors:** Jianjian Wu, Chanzi Liu, Xindi Wang, Chi-Tsun Cheng, Qingfeng Zhou

**Affiliations:** 1 The School of Computer Science and Information Engineering, Hefei University of Technology, Hefei 230601, China; wjj9302@gmail.com; 2 Dongguan Key Lab of Artificial Information Network, Dongguan University of Technology, Dongguan 523808, China; liucz@dgut.edu.cn; 3 Guangdong Provincial Key Laboratory of Intelligent Disaster Prevention and Emergency Technologies for Urban Lifeline Engineering, Dongguan University of Technology, Dongguan 523808, China; 4 The School of Electric Engineering and Intelligentization, Dongguan University of Technology, Dongguan 523808, China; 5 The School of Internet, Anhui University, Hefei 230039, China; xindi.wang@ahu.edu.cn; 6 The Department of Mechanical, Manufacturing, and Mechatronics, RMIT University, Melbourne, VIC 3000, Australia; ben.cheng@rmit.edu.au

**Keywords:** multiple access, sparse-code multiple access (SCMA), blind interference alignment (BIA)

## Abstract

Blind Interference Alignment (BIA) and Sparse Code Multiple Access (SCMA) offer the potential for massive connectivity but face limitations. Our recently proposed Sparsecode-and-BIA-based Multiple Access (SBMA) scheme synergizes their strengths, promising enhanced performance. SBMA leverages flexible user grouping (UG) strategies to effectively manage its unique combination of sparse code constraints and interference alignment requirements, thereby facilitating the fulfillment of diverse Quality of Service (QoS) demands. However, realizing SBMA’s full potential requires efficient joint resource allocation (RA), user scheduling (US), and user grouping (UG). The inherent coupling of these factors within the SBMA framework complicates this task significantly, rendering RA/US solutions designed purely for SCMA or BIA insufficient. This paper addresses this critical open issue. We first formulate the joint RA, US, and UG problems specifically for SBMA systems as an integer optimization task, aiming to maximize the number of users meeting QoS requirements. To tackle this NP-hard problem, we propose an effective algorithm based on Particle Swarm Optimization (PSO), featuring a carefully designed update function tailored specifically for the joint US and UG decisions required in SBMA. Comprehensive simulations demonstrate show that the proposed algorithm significantly outperforms the random-based scheme. Under certain conditions, it serves approximately 280% more users who meet their QoS requirements in high-SNR scenarios.

## 1. Introduction

In wireless communications, Channel State Information (CSI) is essential for effective transmission schemes. In commonly used Orthogonal Frequency Division Multiplexing (OFDM)-based systems, CSI can be estimated using pilot signals allocated to specific subcarriers or slots. At the transmitter, CSI is typically estimated based on channel reciprocity, which necessitates additional uplink pilot transmissions for Transmitter Channel State Information (CSIT). However, the overhead associated with CSIT can be prohibitive, particularly when serving a large number of users. As a result, researchers have focused on developing systems that operate without CSIT [1].

Interference alignment (IA) is a promising solution for multi-user communications [2]. Research has shown that, with full CSI, IA can achieve the optimal Degree of Freedom (DoF). However, the widespread implementation of IA is limited by its reliance on full CSI. To address this issue, scholars have introduced Blind Interference Alignment (BIA) as an alternative [3]. The core concept of BIA involves the use of specific channel patterns and can be divided into two categories: channel-based BIA (CB-BIA) [4] and reconfigurable-antenna-based BIA (RA-BIA) [5]. BIA employs intricately designed channel patterns, often referred to as “supersymbols,” which are extended over several symbol periods. The mode switching operation of reconfigurable antennas, which introduces overhead such as time delays and power consumption, is further considered in [6], where a balance-switching-oriented BIA scheme is proposed. This approach accounts for the mode switching differences among users while maintaining a total switching count comparable to existing methods. While BIA offers a promising solution for multi-user transmission without requiring CSIT, its performance significantly degrades in low Signal-to-Noise Ratio (SNR) regions.

Non-orthogonal multiple access (NOMA) has attracted significant attention from researchers due to its high spectral efficiency and ease of implementation [7,8]. Unlike traditional Orthogonal Multiple Access (OMA) technology, NOMA employs superposition transmission directly for multiple users in the power or code domain. Sparse Code Multiple Access (SCMA) is a promising approach within code-domain NOMA [9]. It utilizes low-density multi-dimensional codebooks and Message Passing Algorithms (MPA) in multi-carrier systems, allowing SCMA to leverage diversity gain across multiple subcarriers as well as unique “shaping gain” derived from its multi-dimensional codebook design [10]. However, the optimal design of codebooks in SCMA remains an open question due to its complexity. In [11], the authors propose a systematic procedure for constructing near-optimal codebooks and introduce a low-complexity algorithm for this purpose. Another critical challenge in SCMA is the development of low-complexity decoding methods. The original MPA involves extensive exponential operations, prompting researchers to adapt it to the logarithm domain to reduce complexity. Variants such as Log-MPA [12] and MAX-Log-MPA [13] have been proposed to alleviate the computational burden.

Recently, Sparsecode-and-Blind Interference Alignment-based Multiple Access (SBMA) was proposed [14], synergizing the benefits of BIA and SCMA to achieve both high diversity and multiplexing gains simultaneously. However, the original SBMA framework outlined in [14] assumes a one-to-one mapping where each ’superuser’ codeword serves only a single physical user. While simplifying the initial analysis, this assumption becomes impractical for systems targeting large numbers of antennas and users, as it demands an excessively long channel coherence time for the necessary alignment procedures. To enhance SBMA’s practicality and overcome this critical limitation, this paper investigates a more flexible model where multiple physical users can share the resources associated with a single SBMA superuser. This approach introduces a crucial user grouping (UG) dimension, allowing the system to operate with fewer active superusers, thereby significantly reducing the required channel coherence time. ﻿

Effectively managing this enhanced SBMA system necessitates the joint optimization of resource allocation (RA), user scheduling (US), and user grouping (UG). While previous work has addressed RA [15] and US [16] individually, and related joint optimization problems have been explored in different contexts such as distributed MIMO systems [17], a dedicated solution for the coupled RA-US-UG challenge specifically within this enhanced SBMA framework remains absent in the literature. This paper fills this gap by formulating the joint RA, US, and UG problem for SBMA, aiming to maximize the number of users served simultaneously while satisfying their QoS requirements. Recognizing the NP-hard nature of this complex optimization task, we propose and develop an effective algorithm based on Particle Swarm Optimization (PSO) tailored to find high-quality solutions. Simulation results are presented to validate the effectiveness and performance benefits of our proposed joint optimization approach. In particular, under certain high-SNR conditions, the proposed algorithm serves up to 280% more users meeting their QoS requirements than the random-based algorithm.

The rest of this paper is organized as follows: Section 2 provides a brief description of the system model. Section 3 introduces the transmitter design of the existing SBMA scheme and analyzes the achievable rate for each user. Section 4 presents the joint resource allocation and user scheduling problem within the SBMA system, followed by the proposal of a PSO-based solution. Section 5 presents simulation results comparing the PSO-based algorithm with a random-based algorithm, demonstrating the efficiency of the proposed approach. Finally, Section 6 concludes the paper.

## 2. System Model

Consider a downlink multi-user system consisting of a base station (BS) and multiple users within a designated area. The BS is equipped with $N_{t}$ transmit antennas, while all users are equipped with reconfigurable antennas that can adjust their operational mode. The reconfigurable antenna provides new signal degrees of freedom for the receiving users, enabling controllable interference management, which is achieved through the BIA technique. The area is divided into *N* clusters, and without loss of generality, we assume that each cluster covers the same total number of users, denoted by *K*. Note that user clusters are pre-grouped based on their physical locations, where users in close proximity are assigned to the same cluster. Typical scenarios include indoor environments with multiple rooms. The system operates with *J* available subcarriers, and we assume there is no CSIT.

The BS is tasked with allocating $A_{n}$ transmit antennas and $J_{n}$ subcarriers for the *n*-th cluster to serve as many users as possible. This paper focuses on a spatially correlated Multiple-Input Multiple-Output (MIMO) channel model [18], where the channel model between any selected $A_{n}$ transmit antennas and the *k*-th user is modeled as follows:

(1) $$H_{k}^{A_{n}}=H_{iid-k}^{A_{n}}\sqrt{R_{TX}^{A_{n}}}.$$

$H_{iid-k}^{A_{n}}$ contains independent and identically distributed complex zero-mean, unit variance, Gaussian random entries, while $R_{TX}^{A_{n}}$ is the antenna correlation matrix of the BS. In $R_{TX}^{A_{n}}$, the element $r_{ij}$ at the *i*-th row and the *j*-th column is the correlation coefficient between the *i*-th and the *j*-th antenna, which is modeled as

(2) $$r_{ij}=J_{0}\left(\frac{2\pi d_{ij}}{\lambda }\right),$$

where $d_{ij}$ is the distance between two antennas, λ is the carrier wavelength, and $J_{0}\left(\cdot \right)$ denotes the zeroth-order Bessel function of first kind. Note that we consider one antenna at users, the correlation matrix of the multiple receive antennas is omitted.

## 3. SBMA

Given the absence of CSIT in the system, implementing traditional beamforming schemes for serving multiple users becomes challenging. BIA and SCMA present two potential solutions for systems lacking CSIT. However, both approaches have their limitations. For example, BIA struggles with significant decoding time delays and constraints related to channel coherence time. Conversely, SCMA is affected by high decoding complexity, security vulnerabilities, and a lack of methods for achieving spatial advantages.

To address these limitations, a novel scheme called SBMA has been proposed [14]. It has been shown that SBMA effectively combines the strengths of SCMA and BIA while mitigating their drawbacks. This paper focuses on joint resource allocation and user scheduling within the context of SBMA.

### 3.1. SBMA Transmitter

Since this paper focuses on the joint resource allocation and user scheduling, we will provide a brief overview of the transmitter design for SBMA in this section.

**Figure 1:**
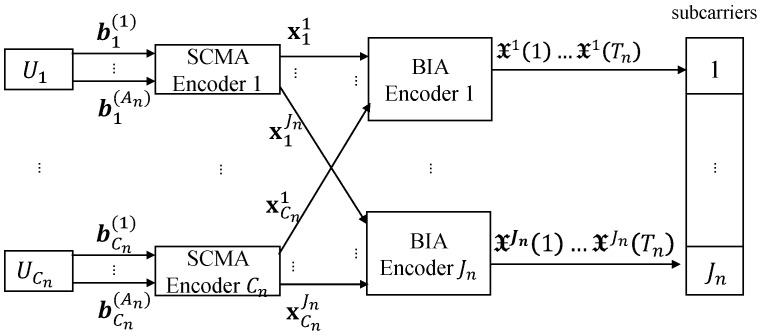
Transmitter design for the *n*-th cluster.

Figure 1 illustrates the transmitter design for the *n*-th cluster. We define $U_{k}$ as the *k*-th superuser, where $1\leq k\leq C_{n}$, with $C_{n}$ representing the number of superusers in the *n*-th cluster. A superuser $U_{k}$ can either represent a single user, as assumed in [14], or comprise a group of multiple users. The encoding process involves two steps: SCMA encoding and BIA encoding.

#### 3.1.1. SCMA Encoding

Consider a transmitting binary vector $b_{k}^{\left(n_{t}\right)}\in B^{L\times 1}$ at the $n_{t}$-th transmit antenna, where $1\leq n_{t}\leq A_{n}$. The value of *L* depends on the number of available subcarriers, $J_{n}$, and the predetermined degree of resource reuse. The transmitting data $b_{k}^{\left(n_{t}\right)}$ can be allocated to multiple users in the *k*-th superuser.

Let $b_{k}^{\left(n_{t}\right)}\left(l\right)$ denote the *l*-th element of $b_{k}^{\left(n_{t}\right)}$. After the SCMA encoding, $b_{k}^{\left(n_{t}\right)}\left(l\right)$ is mapped into a codeword $s_{kl}^{\left(n_{t}\right)}$, which is superposed over $J_{n}$ subcarriers. This process produces the transmitting symbol vector $S_{k}^{\left(n_{t}\right)}=\sum_{l=1}^{L}s_{kl}^{\left(n_{t}\right)}\in C^{J_{n}}$. Denote $S_{k}^{j(n_{t})}$ as the *j*-th element in $S_{k}^{\left(n_{t}\right)}$. Furthermore, we focus on each subcarrier and denote $x_{k}^{j}={\left[S_{k}^{j(1)}\;\cdots S_{k}^{j(A_{n})}\right]}^{T}$ as the corresponding transmitting vector on the *j*-th subcarrier. Thus, if $A_{n}=2$, we have $x_{k}^{j}={\left[S_{k}^{j(1)}\;S_{k}^{j(2)}\right]}^{T}$.

#### 3.1.2. BIA Encoding

BIA encoding is then applied at each subcarrier by employing symbol extension in the time domain [3,14]. BIA achieves interference-free transmission without the need for CSIT by leveraging specific channel conditions, referred to as channel patterns. Using reconfigurable antennas to artificially change the channel state allows for the reconstruction of required channel patterns, known as “supersymbols”. Additionally, a simple transmitting beamforming vector and a decoding matrix are designed for each user.

Assuming $A_{n}=2$, $C_{n}=6$, the supersymbols and transmitting beamforming vectors $V_{k}$ on the *j*-th subcarrier are shown in Table 1 and Table 2, respectively, where “slot” represents the basic waveform period in wireless networks. Note that in Table 2, 0 denotes an all-zero matrix with a size of 2×2. Without loss of generality, consider the *j*-th subcarrier at the first superuser, the encoded transmitting signal can be denoted as

(3) $$\begin{array}{cc} X^{j} & ={\left[\begin{array}{ccc} X^{j}\left(1\right) & \cdots & X^{j}\left(7\right) \end{array}\right]}^{T}=V_{1}x_{1}^{j}+V_{2}x_{2}^{j}+V_{3}x_{3}^{j}+V_{4}x_{4}^{j}+V_{5}x_{5}^{j}+V_{6}x_{6}^{j}\\ \; & =\left[\begin{array}{c} x_{1}^{j}+x_{2}^{j}+x_{3}^{j}+x_{4}^{j}+x_{5}^{j}+x_{6}^{j}\\ x_{1}^{j}\\ x_{2}^{j}\\ x_{3}^{j}\\ x_{4}^{j}\\ x_{5}^{j}\\ x_{6}^{j} \end{array}\right] \end{array},$$

where $X^{j}\left(t\right)\in C^{A_{n}\times 1}$ denotes the transmitting vector from the BS in the *j*-th subcarrier at the *t*-th slot.

Further, the received signal at the *j*-th subcarrier can be expressed as

(4) $$\begin{array}{c} y_{1}^{j}={\left[\begin{array}{cccc} y_{1}^{j}\left(1\right) & y_{1}^{j}\left(2\right) & \cdots & y_{1}^{j}\left(7\right) \end{array}\right]}^{T}=\left[\begin{array}{cccc} h_{1}^{j}\left(1\right) & 0 & \cdots & 0\\ 0 & h_{1}^{j}\left(2\right) & \cdots & 0\\ \vdots & \vdots & \ddots & 0\\ 0 & 0 & 0 & h_{1}^{j}\left(1\right) \end{array}\right]\left[\begin{array}{c} X^{j}\left(1\right)\\ \vdots \\ X^{j}\left(7\right) \end{array}\right]+Z_{1}^{j} \end{array}$$

Here $Z_{1}^{j}={\left[\begin{array}{ccc} z_{1}^{j}\left(1\right) & \cdots & z_{1}^{j}\left(7\right) \end{array}\right]}^{T}$ is the AWGN vector over seven slots and 0 denotes a all-zero matrix with the size of 1×2.

#### 3.1.3. Achievable Rate

Given the absence of CSIT, we assume that the BS allocates the same power $P_{t}$ to each cluster. The transmitting power is evenly distributed among superusers within each cluster. Previous studies have established the achievable rates for BIA [3] and SBMA [14] under these conditions. Therefore, we can derive the rates for users in the SBMA system.

Let $K_{n,c}$ denote the number of simultaneously served users in the *c*-th superuser at the *n*-th cluster. Suppose the transmitting power for each cluster is then equally allocated among $K_{n,c}$ users. The achievable rate of the $k^{*}$-th user in the $c_{n}$-th superuser at the *n*-th cluster is

(5) $$\begin{array}{c} R_{nk^{*}}=\frac{1}{\alpha_{n}K_{n,c}}\sum_{j=1}^{J_{n}}E\left[\log\det\left(I+\frac{\alpha_{n}\gamma }{{\left(A_{n}\right)}^{2}C_{n}}H_{k^{*}j}^{A_{n}}\;H_{k^{*}j}^{A_{n}†}\right)\right] \end{array},$$

where $\gamma =P_{t}/\sigma^{2}$ is Signal-to-Noise Ratio (SNR), $\alpha_{n}=A_{n}+C_{n}-1$ is the normalization coefficient considering the length of total slots needed for SBMA, and

(6) $$H_{k^{*}j}^{A_{n}}=\left[\begin{array}{c} \frac{1}{C_{n}}h_{k^{*}}^{j}\left(1\right)\\ \cdots \\ h_{k^{*}}^{j}\left(A_{n}\right) \end{array}\right]$$

is the constructed channel matrix of allocated $A_{n}$ antennas.

## 4. Problem Formulation and the Proposed Algorithm

SBMA effectively combines the advantages of BIA and SCMA, addressing their respective drawbacks. However, previous studies did not consider user scheduling and grouping within superusers. This paper primarily focuses on the joint resource allocation, user scheduling and grouping problem within an SBMA system.

The problem can be described as follows: In a wireless network with *N* clusters covered by a BS, the BS must allocate antennas and subcarriers for each cluster. Additionally, by implementing SBMA, the BS needs to develop user scheduling and grouping strategies within the clusters. The objective of the BS is to serve as many users as possible. Given the absence of accurate CSIT, the BS allocates transmit power equally among clusters and users.

**Figure 2:**
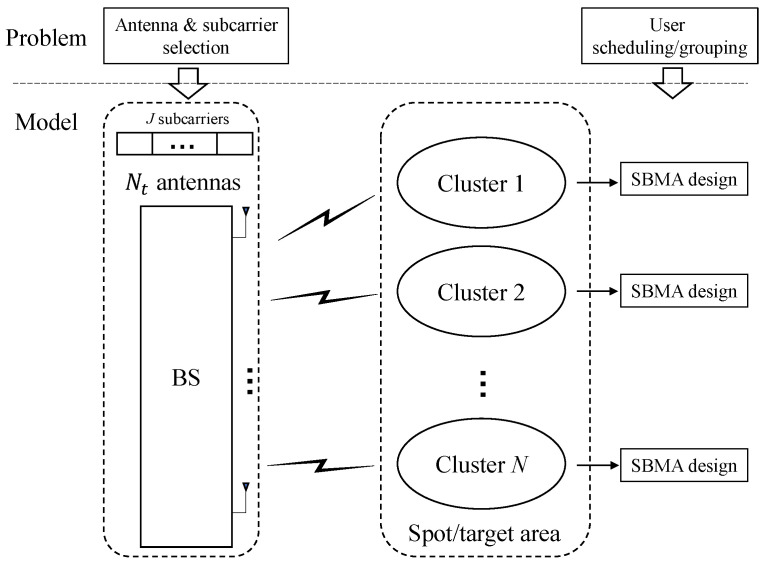
System model and the problem.

To facilitate understanding, Figure 2 illustrates the overall system model. Note that antenna and subcarrier selection significantly influence performance across clusters, while user scheduling and grouping are closely tied to the performance within each individual cluster. These problems can be addressed using an alternating optimization framework; however, the convergence behavior of alternating optimization is often not well understood. Therefore, in this paper, we jointly optimize these problems and propose an efficient PSO-based algorithm.

### 4.1. Problem Formulation

To formulate the problem, we give some definitions as follows:

1. a is of size $1\times N_{t}$, and the values range from 1 to *N*. The $n_{t}$-th element, $a_{n_{t}}$, denotes that the $n_{t}$-th antenna is allocated for the $a_{n_{t}}$-th cluster.
2. s is of size 1×J, and the values range from 1 to *N*. The *j*-th element, $s_{j}$, denotes that the *j*-th subcarrier is allocated for the $s_{j}$-th cluster.
3. $u_{n}$ is of size 1×K, and the values range from 0 to *K*. The *k*-th element, $g_{n}^{k}$, denotes that the *k*-th user in the *n*-th cluster is scheduled into the $g_{n}^{k}$-th superuser. Note that if $g_{n}^{k}=0$, the user is not served by the BS. Additionally, $g_{n}^{i}=g_{n}^{j}\neq 0$ means that the *i*-th and the *j*-th user are scheduled and grouped into a superuser in SBMA.
4. Furthermore, we denote $C_{n}$ as the number of superusers in the *n*-th cluster, and $K_{n,c}$ is the number of users in the *c*-th superuser.

The main objective of this paper is to develop an algorithm for the BS to maximize user serviceability. Utilizing the previously defined parameters, resource allocation can be optimized by focusing on the antenna allocation, represented by a, and the subcarrier allocation, represented by s. Additionally, user scheduling and grouping can be achieved by optimizing $u_{n}$. Therefore, we can formulate the problem as follows:

(7) $$\begin{array}{cc} \; & \max_{a,s,u_{n}}\sum_{n}\sum_{n}K_{n,c}\\ s.t.\;\; & C1:R_{nk}\geq R_{min},\forall u_{n}\neq 0,\\ \; & C2:find(a=n)\geq 2,\forall n=1\cdots N,\\ \; & C3:find(s=n)\geq 2,\forall n=1\cdots N,\\ \; & C4:C_{n}\geq 2, \end{array}$$

where C1 is the key QoS constraint, ensuring a minimum served rate for all active users. C2−C3 arise from the SCMA and BIA system requirements for multi-antenna, multi-carrier, and multi-user operation.

The problem presented in Equation (7) defines a discrete combinatorial optimization challenge. The integer assignments of antennas, subcarriers, and users to superusers create an exponential search space, which renders it NP-hard. To address this issue effectively, we propose a PSO-based algorithm.

### 4.2. PSO-Based Algorithm

PSO is a swarm intelligence-based optimization algorithm that emulates the cooperative and competitive behaviors observed in bird flocks or fish schools [19]. It seeks the optimal solution by simulating particle movement and information sharing within a swarm. In the context of PSO, particles symbolize potential solutions, and the entire swarm represents the solution space. Particle positions and velocities are updated based on the knowledge of the best-known solutions at the individual level ($p_{\mathrm{best}}$) and the global level ($g_{\mathrm{best}}$). A fitness function is employed to evaluate each solution.

During each iteration, the fitness value of each particle’s current position updates $p_{\mathrm{best}}$, the fitness value of the entire swarm’s current position updates $g_{\mathrm{best}}$, and the positions and velocities of each particle are adjusted based on $p_{\mathrm{best}}$ and $g_{\mathrm{best}}$. Traditional PSO is not well-suited for discrete optimization problems, leading to the development of Discrete PSO (DPSO) [20]. In DPSO, particle positions are discretized and commonly represented using binary or integer encoding. The update formulas for velocity and position undergo modifications to accommodate discrete problems. While the fundamental concept of DPSO aligns with traditional PSO, adjustments are made to the position and velocity updates to handle discrete values and domain-specific encoding schemes.

To solve problem (7) using PSO, we need to give definitions of position and velocity, as well as their updating operations and the fitness function, which are summed as followed.

1. **Position**: The position of the *i*-th swarm is defined as
  (8) $$X_{i}=\left[\begin{array}{ccccc} a & s & u_{1} & \cdots & u_{N} \end{array}\right].$$
  **Constraints**: (1) Suppose all clusters are allocated at least two antennas (SBMA is not capable for only one antenna available). Thus, in a, each number from 1 to *N* appears at least twice. (2) Each cluster needs to be allocated at least 2 subcarriers. Thus, in s, each number from one to *N* appears at least twice. (3) There are at least two superusers in each cluster. Thus, in $u_{N}$, there are at least two different non-zero values.
2. **Velocity**: The velocity of the *i*-th swarm relative to the *j*-th swarm is
  (9) $$\begin{array}{cc} v_{i}= & \left[v_{i}^{a}\;v_{i}^{s}\;v_{i,1}^{u}\cdots v_{i,N}^{u}\right]=Vel\left(X_{i},X_{j}\right)\\ = & \left[f_{1}\left(a_{i},a_{j}\right)\;\;f_{1}\left(s_{i},s_{j}\right)\;\;f_{2}\left(u_{i,1},u_{j,1}\right)\;\;\cdots \;\;f_{2}\left(u_{i,N},u_{J,N}\right)\right] \end{array},$$
  where $v_{i}^{a}$ and $v_{i}^{s}$ are velocity vectors corresponding to a and s of the *i*-th swarm, respectively; $v_{i,n}^{u}$ is the velocity vector correspondinig to $u_{i,n}$; $u_{i,n}$ is the corresponding user schedue and grouping vector of the *i*-th swarm, and is defined the same as $u_{n}$ in Section 4.1; Vel(∗) is the computing function of velocity and is composed with two sub-functions $f_{1}\left(*\right)$ and $f_{2}\left(*\right)$. The operation of $f_{1}\left(*\right)$ and $f_{2}\left(*\right)$ is described as follows:
  $x_{o}=f_{1}\left(x_{i},x_{j}\right)$ is defined such that if $x_{i}$ and $x_{j}$ share the same value, the corresponding position in $x_{o}$ is set to 0; conversely, if $x_{i}$ and $x_{j}$ have distinct values, the corresponding position in $x_{o}$ adopts the value of $x_{j}$.
  $x_{o}=f_{2}\left(x_{i},x_{j}\right)$: $f_{2}$ closely resembles $f_{1}$ with the addition of an extra operation. When $x_{i}$ and $x_{j}$ differ in value, the corresponding position in $x_{o}$ is determined by adding one to the value of $x_{j}$. This design choice is motivated by the value range of $u_{i,n}$, which spans from 0 to *K*. Introducing 0 in v is necessary to denote an unchanged operation.
3. **Update of Velocity**: In the iteration process, we define a updating function for velocity of each swarm as
  (10) $$v_{o}={\mathrm{update}}_{V}\left(w,v_{0},c_{1},v_{p},c_{2},v_{g}\right),$$
  where *w* is the inertia weight, $c_{1}$ and $c_{2}$ are predefined learning coefficient in PSO, $v_{0}$ is the current velocity of the swarm while $v_{p}$ is the velocity according to the individual best-known swarm, and $v_{g}$ is the velocity according to the global best-known swarm. Note that the index of the swarm is omitted here for ease of reading.
  The operation of ${\mathrm{update}}_{V}\left(w,v_{0},c_{1},v_{p},c_{2},v_{g}\right)$ is defined as follows: The probability of a value being 0 in $v_{o}$ is denoted as
  (11) $$p_{0}=\left(1-w\right)\left(1-c_{1}\right)\left(1-c_{2}\right);$$
  the probability of a value in $v_{o}$ being the same as corresponding value in $v_{0}$ is denoted as
  (12) $$p_{1}=\frac{w}{w+c_{1}+c_{2}}\left(1-\left(1-w\right)\left(1-c_{1}\right)\left(1-c_{2}\right)\right);$$
  the probability of a value in $v_{o}$ being the same as corresponding value in $v_{p}$ is denoted as
  (13) $$p_{2}=\frac{c_{1}}{w+c_{1}+c_{2}}\left(1-\left(1-w\right)\left(1-c_{1}\right)\left(1-c_{2}\right)\right);$$
  the probability of a value in $v_{o}$ being the same as corresponding value in $v_{g}$ is denoted as
  (14) $$p_{3}=\frac{c_{2}}{w+c_{1}+c_{2}}\left(1-\left(1-w\right)\left(1-c_{1}\right)\left(1-c_{2}\right)\right).$$
4. **Update of Position**: In the iteration process, we define a updating function for positions of each swarm as
  (15) $$\begin{array}{cc} X_{o}= & \left[\begin{array}{ccccc} a_{o} & s_{o} & u_{o,1} & \cdots & u_{o,N} \end{array}\right]={\mathrm{update}}_{X}\left(X,v\right)\\ = & [upd_{1}\left(a,v^{a}\right)\;upd_{1}\left(s,v^{s}\right)\;upd_{2}\left(u_{1},v_{1}^{u}\right)\cdots upd_{2}\left(u_{N},v_{N}^{u}\right)], \end{array}$$
  where X is the current positon; v is the updated velocity; the function $upd_{1}\left(*\right)$ and $upd_{2}\left(*\right)$ are updating functions for a, s and u. The index of the swarm is also omitted here, e.g., for the *i*-th swarm we have $v^{a}=v_{i}^{u}$, $v^{s}=v_{i}^{s}$, and $v_{n}^{u}=v_{i,n}^{u}$. The operation of $upd_{1}\left(*\right)$ and $upd_{2}\left(*\right)$ is described as follows:
  $a_{o}=upd_{1}\left(a,v^{a}\right)$: If any position in $v^{a}$ is assigned 0, the corresponding positions in $a_{o}$ are assigned the same as the values in a. Furthermore, other positions in $a_{o}$ are assigned the corresponding non-zero values in $v^{a}$. After these operation, if $a_{o}$ does not meet the constraints that we defined before, some random positions in $a_{o}$ needs to be reassigned.
  $u_{o}=upd_{2}\left(u,v^{u}\right)$: $upd_{2}$ is similar to $upd_{1}$, with an additional operation. The non-zero values in $v^{u}$ needs to be subtracted by 1 before assigned to corresponding positions in $u_{o}$.
5. **Fitness function**: Since the objective of the optimization problem is to maximize the overall served users, we define the fitness function as
  (16) $$F\left(X\right)=K^{s},$$
  where $K^{s}$ is the number of served users that meet the lowest rate constraint in the system by applying the joint resource allocation and user schedulling principle according to X.

According to the above definitions, the details of the proposed algorithm are shown in Algorithm 1. Once the optimal RA, US, and UG solution is found by the proposed algorithm, a valid SBMA transmission scheme that meets the BIA conditions can be constructed based on our previous work [14].
**Algorithm 1** PSO-based algorithm for SBMA**Input:** Position $X_{i}$ and velocity $v_{i}$ for the *i*-th particle; the number of iteration *M*; the number of particles *G*; the maximum inertia weight $w_{max}$, the minimum inertia weight $w_{min}$, learning coefficients $c_{1}$ and $c_{2}$.**Output:** Solution $X_{o}$   1:Initialize $X_{i}$, $v_{i}$ and $p_{\mathrm{best}}^{i}$ for each particle.   2:Find the globally best position $g_{\mathrm{best}}$.   3:**while** m<M **do**   4:    $w=w_{max}-\left(w_{max}-w_{min}\right)m/M;$   5:    **for** i=1,2,⋯,G **do**   6:      Update the velocity according to (10).   7:      Update the position according to (15).   8:      Obtain the fitness value according to (16).   9:      Update the locally best position $p_{\mathrm{best}}^{i}$. 10:    **end for** 11:    Update $g_{\mathrm{best}}$. 12:**end while** 13:$X_{o}=g_{\mathrm{best}}$.

## 5. Simulation

To validate the proposed algorithm, we conduct simulations in this section.

To the best of our knowledge, there are no well-established solutions for the studied joint RA, US, and UG problem. Although SCMA is integrated into the SBMA framework, we do not compare with a standalone SCMA system because SCMA acts as a component rather than a counterpart of SBMA. Moreover, the joint optimization of RA, US, and UG in SBMA introduces additional complexity not present in traditional SCMA settings, making a direct comparison less meaningful. Therefore, we present the random-based algorithm as a baseline for comparison. The random-based algorithm generates random integer solutions for the optimization problem (7). For fair comparison, results from this baseline are averaged over multiple independent runs.

The system configuration is described as follows: $N_{t}=20$, N=5, K=20, J=20, and we assume each cluster is allocated four subcarriers. The parameter settings of the proposed algorithm are: M=70 and G=100.

**Figure 3:**
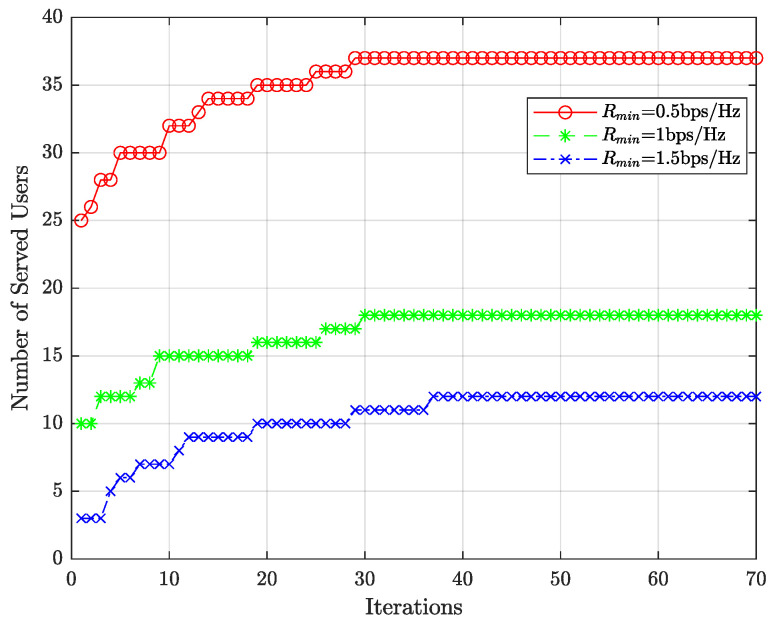
Number of served users (meet the lowest rate $R_{min}$) vs. iterations when SNR =2 dB.

Figure 3 shows the number of served users achieved by the proposed algorithm through iterations when the SNR is 2 dB. The figure indicates that the performance curves almost converge after 30 iterations, which is efficient in practical terms. Furthermore, with improved minimum rate ($R_{min}$), the number of served users decreases.

**Figure 4:**
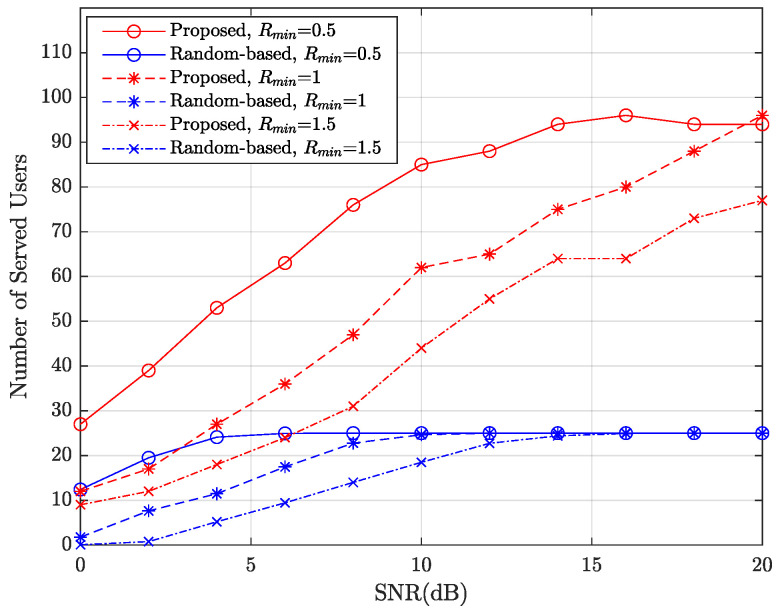
Number of served users (meet the lowest rate $R_{min}$) vs. SNR.

Figure 4 shows the performance of the proposed algorithm and the random-based algorithm as a function of the SNR. It is evident that as the SNR increases, the proposed algorithm consistently approaches the maximum number of served users (NK=100), while the random-based algorithm reaches a smaller number (approximately 25). Specifically, in high SNR regions (SNR > 28 dB), the proposed algorithm can support approximately 280% more users when $R_{min}\leq 1$. Furthermore, for smaller $R_{min}$ values, both algorithms exhibit better performance and faster convergence.

**Figure 5:**
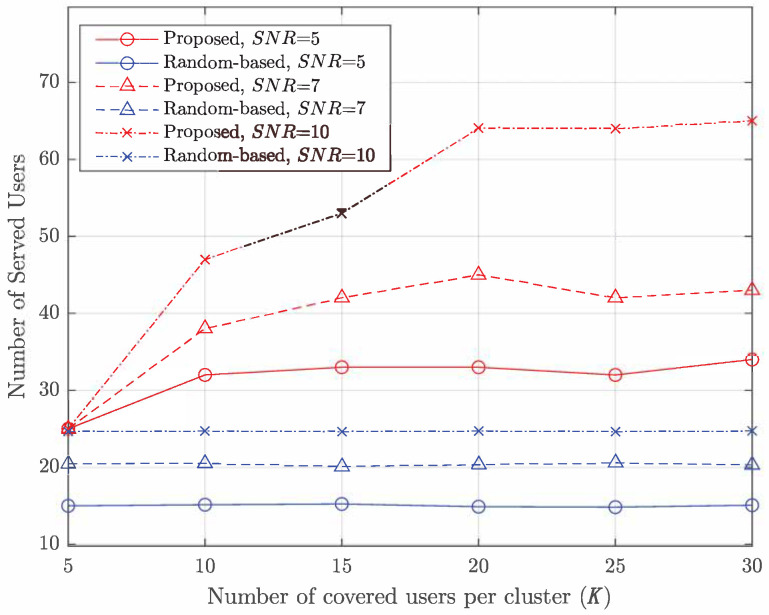
Number of served users (meet the lowest rate $R_{min}=1$) vs. *K*.

Figure 5 illustrates the performance as a function of the number of users per cluster (*K*). It shows that the proposed algorithm improves as *K* increases, while the performance of the random-based algorithm remains unaffected by *K*. Furthermore, the proposed algorithm serves approximately 113% more users than the random-based algorithm at an SNR of 5 dB, 115% more at an SNR of 7 dB, and 160% more at an SNR of 10 dB. At higher SNRs, the proposed algorithm achieves significantly better performance, particularly in scenarios where a large number of users are covered in each cluster.

## 6. Conclusions

In this paper, we addressed the critical challenge of resource management in the novel SBMA multiple access system. We formulated the problem as a joint optimization of resource allocation, user scheduling, and grouping, capturing the unique interplay between these elements in SBMA. We proposed an effective PSO-based algorithm specifically designed to handle this complex, NP-hard integer programming task, where the PSO search intrinsically determines compatible user groups alongside scheduling and resource assignments. Simulation results confirmed that our joint optimization approach significantly enhances the number of satisfied users compared to random-based methods, demonstrating the importance of simultaneously considering RA, US, and UG for efficient SBMA operation.

While the proposed PSO-based algorithm demonstrates high efficiency and strong performance in the evaluated scenarios, its scalability to large-scale systems remains a challenge. Therefore, developing more efficient and higher-performance algorithms becomes essential. In this context, advanced heuristic approaches, such as carefully designed greedy algorithms and other intelligent algorithms, offer a promising direction by potentially achieving near-optimal solutions with lower computational overhead.

## Figures and Tables

**Table 1:** Supersymbol of BIA.

	Slot-1	Slot-2	Slot-3	Slot-4	Slot-5	Slot-6	Slot-7
$U_{1}$	$h_{1}^{j}\left(1\right)$	$h_{1}^{j}\left(2\right)$	$h_{1}^{j}\left(1\right)$	$h_{1}^{j}\left(1\right)$	$h_{1}^{j}\left(1\right)$	$h_{1}^{j}\left(1\right)$	$h_{1}^{j}\left(1\right)$
$U_{2}$	$h_{2}^{j}\left(1\right)$	$h_{2}^{j}\left(1\right)$	$h_{2}^{j}\left(2\right)$	$h_{2}^{j}\left(1\right)$	$h_{2}^{j}\left(1\right)$	$h_{2}^{j}\left(1\right)$	$h_{2}^{j}\left(1\right)$
$U_{3}$	$h_{3}^{j}\left(1\right)$	$h_{3}^{j}\left(1\right)$	$h_{3}^{j}\left(1\right)$	$h_{3}^{j}\left(2\right)$	$h_{3}^{j}\left(1\right)$	$h_{3}^{j}\left(1\right)$	$h_{3}^{j}\left(1\right)$
$U_{4}$	$h_{4}^{j}\left(1\right)$	$h_{4}^{j}\left(1\right)$	$h_{4}^{j}\left(1\right)$	$h_{4}^{j}\left(1\right)$	$h_{4}^{j}\left(2\right)$	$h_{4}^{j}\left(1\right)$	$h_{4}^{j}\left(1\right)$
$U_{5}$	$h_{5}^{j}\left(1\right)$	$h_{5}^{j}\left(1\right)$	$h_{5}^{j}\left(1\right)$	$h_{5}^{j}\left(1\right)$	$h_{5}^{j}\left(1\right)$	$h_{5}^{j}\left(2\right)$	$h_{5}^{j}\left(1\right)$
$U_{6}$	$h_{6}^{j}\left(1\right)$	$h_{6}^{j}\left(1\right)$	$h_{6}^{j}\left(1\right)$	$h_{6}^{j}\left(1\right)$	$h_{6}^{j}\left(1\right)$	$h_{6}^{j}\left(1\right)$	$h_{6}^{j}\left(2\right)$

**Table 2:** Beamforming vectors of BIA.

$U_{1}$	$V_{1}={\left[\;I\;I\;0\;0\;0\;0\;0\;\right]}^{T}$
$U_{2}$	$V_{2}={\left[\;I\;0\;I\;0\;0\;0\;0\;\right]}^{T}$
$U_{3}$	$V_{3}={\left[\;I\;0\;0\;I\;0\;0\;0\;\right]}^{T}$
$U_{4}$	$V_{4}={\left[\;I\;0\;0\;0\;I\;0\;0\;\right]}^{T}$
$U_{5}$	$V_{5}={\left[\;I\;0\;0\;0\;0\;I\;0\;\right]}^{T}$
$U_{6}$	$V_{6}={\left[\;I\;0\;0\;0\;0\;0\;I\;\right]}^{T}$

## Data Availability

The original contributions presented in this study are included in the article. Further inquiries can be directed to the corresponding author.
